# Nanocarrier System Based on Jabuticaba Peel Co-Products and Caffeine for Photoprotective and Regenerative Skin Applications

**DOI:** 10.3390/ph19101517

**Published:** 2026-09-25

**Authors:** Thalita do Espírito Santo Alves, Leonardo Froes de Azevedo Chang, Tathyana Benetis Piau, Isadora Florêncio de Souza, Caio Leal, Marina Arantes Radicchi, Danielle Galdino de Souza, Cesar Koppe Grisolia, Urska Vrhovsek, Eduardo Antonio Ferreira, Eliana Fortes Gris, Sônia Nair Báo, Victor Carlos Mello

**Affiliations:** 1 Cooil Cosmetics Ltd., Brasília 70910-900, DF, Brazil; 2 Faculty of Health Sciences and Technologies, Campus Ceilandia, University of Brasília, Ceilandia, Brasília 72220-275, DF, Brazil; 3 Department of Cell Biology, Institute of Biological Sciences, University of Brasília, Brasília 70297-400, DF, Brazilsnbao@unb.br (S.N.B.); 4 Laboratory of Genetic Toxicology, Department of Genetics and Morphology, Institute of Biological Sciences, University of Brasília, Brasília 70910-900, DF, Brazil; tathyanabenetis@gmail.com (T.B.P.);; 5 Nanobiotechnology Laboratory, Genetics & Morphology Department, Institute of Biological Sciences, University of Brasília, Brasília 70910-900, DF, Brazil; danielle.galdino@hotmail.com; 6 Edmund Mach Foundation, Via E. Mach 1, 38010 San Michele all’Adige, Italy; 7 Center for Metabolomics, Biotechnical Faculty, University of Ljubljana, Jamnikarjeva 101, 1000 Ljubljana, Slovenia; 8 Graduate Program in Health Sciences and Technologies, Faculty of Health Sciences and Technologies, Campus Ceilandia, University of Brasília, Ceilandia, Brasília 72220-275, DF, Brazil

**Keywords:** polyphenols, photoprotection, UV-induced oxidative stress, circular bioeconomy, zebrafish ecotoxicity

## Abstract

**Background**: Ultraviolet (UV) radiation, urban pollution and depletion of cutaneous antioxidant reserves are interconnected drivers of photoaging, matrix metalloproteinase (MMP) activation, mitochondrial dysfunction and dermal extracellular matrix degradation. Plant polyphenols can counteract these processes, but topical efficacy is limited by chemical instability, polarity-driven partitioning and a hostile stratum corneum delivery interface. Here we report a green nanostructured lipid carrier co-loading a phenolic-rich jabuticaba (*Plinia cauliflora*) peel extract and caffeine (NLC-JC), engineered with Amazonian lipids (*Astrocaryum murumuru* butter and *Orbignya phalerata* oil) and stabilized by a Natural Deep Eutectic Solvent (NaDES)-like microenvironment. **Methods**: UPLC profiling was used to determine the polyphenolic composition of the extract. Physicochemical characterization included hydrodynamic diameter and PDI, ζ-potential, lipid matrix organization (XRD) and morphology (TEM), as well as colloidal stability over 30 days. Cytotoxicity was assessed in SH-4 melanocytes (ISO 10993-5) and in zebrafish (Danio rerio) embryos (96-h IC_50_, GHS acute hazard threshold). Functional antioxidant and photoprotective activity was evaluated by DPPH radical scavenging, and by attenuation of UVC- and H_2_O_2_-induced anion superoxide generation, alongside assessment of cellular and nuclear morphology under combined oxidative stress. **Results**: UPLC profiling revealed a polyphenolic fingerprint dominated by ellagic acid (476.8 ± 19.7 µg g^−1^), quercetin-3-rhamnoside (90.9 ± 4.6 µg g^−1^), quercetin (83.7 ± 1.4 µg g^−1^), cyanidin-3-glucoside (73.0 ± 1.2 µg g^−1^) and protocatechuic acid (68.2 ± 2.2 µg g^−1^), bioactives with documented anti-MMP, NF-κB-suppressing and ROS-scavenging activities. Optimized NLC-JC showed a hydrodynamic diameter of 56.1 nm (PDI = 0.131), negative ζ-potential, amorphous lipid matrix (XRD) and spherical morphology (TEM, ≈150–200 nm electron-dense particles), with 30-day colloidal stability. NLC-JC exhibited low cytotoxicity in SH-4 melanocytes (IC_50_ = 2304 μg mL^−1^), preserving ≥70% viability across the cosmetically relevant range (ISO 10993-5), and a 96-h LC_50_ of 2013 μg mL^−1^ in zebrafish (*Danio rerio*) embryos, three orders of magnitude above the GHS acute hazard threshold (100 mg L^−1^). Functionally, NLC-JC produced ≈50% DPPH radical scavenging at concentrations comparable to ascorbic acid, attenuated UVC- and H_2_O_2_-induced anion superoxide generation and preserved cellular and nuclear morphology under combined oxidative stress. **Conclusion**: These data position NLC-JC as a safe-by-design, multifunctional dermocosmetic platform combining agro-industrial valorization with mechanism-anchored photoprotection.

## 1. Introduction

The growing demand for sustainable technologies has stimulated the development of innovative strategies that combine technological performance, environmental responsibility, and biodiversity valorization. Agro-industrial by-products have emerged as valuable sources of bioactive compounds for cosmetic and pharmaceutical applications [1,2].

Jabuticaba (*Plinia cauliflora*) peel is an underutilized residue rich in phenolic compounds, anthocyanins, and tannins with recognized antioxidant activity, while caffeine exhibits antioxidant, anti-inflammatory, and photoprotective properties. However, the instability and limited bioavailability of these compounds can restrict their practical application [3,4,5].

Photoaging of human skin is a multifactorial degenerative process driven primarily by chronic exposure to ultraviolet (UV) radiation. At the molecular level, UVA reaches the dermis, generating reactive oxygen species (ROS) via riboflavin- and porphyrin-mediated photosensitization, whereas UVB is preferentially absorbed by epidermal DNA, producing cyclobutane pyrimidine dimers (CPDs) and 6-4 photoproducts [6]. Both wavelengths converge on the activation of mitogen-activated protein kinase (MAPK) cascades, AP-1 and NF-κB signaling, upregulating MMP-1, MMP-3 and MMP-9—the collagenolytic, stromelytic and gelatinolytic enzymes responsible for dermal extracellular matrix degradation, wrinkle formation and loss of skin biomechanical integrity [6,7]. Concurrent oxidative damage to mitochondrial DNA, peroxidation of cellular lipids and apoptotic activation of p53 amplify the senescent dermal phenotype and increase carcinogenic risk [7].

Topical antioxidant strategies are central to modern dermocosmetic design, yet conventional polyphenol formulations face three structural limitations: (i) chemical instability of majority phenolic compounds against oxygen, light and pH fluctuations; (ii) limited percutaneous absorption owing to molecular polarity and the lipophilic barrier function of the stratum corneum; and (iii) low sustained-release performance, leading to peak-and-trough exposure profiles that may trigger pro-oxidant hormetic responses at supratherapeutic doses [8,9]. Second-generation nanostructured lipid carriers (NLCs) bypass these constraints by combining solid and liquid lipid phases that create defect-rich amorphous matrices with high loading capacity, biocompatibility with the skin acid mantle, occlusive hydration properties, and efficient intercellular and transfollicular penetration pathways [10].

The biological basis for co-encapsulating jabuticaba peel polyphenols and caffeine rests on a complementary mechanistic axis: polyphenols neutralize ROS at the lipid-water interface and modulate MMP/NF-κB pathways, while caffeine contributes water-phase radical scavenging, mild UV absorption and adenosine receptor-mediated cytoprotection [7,9,11]. We hypothesized that an Amazonian lipid NLC co-loaded with jabuticaba peel polyphenols and caffeine (NLC-JC) would: (i) generate sub-200 nm, monodisperse, negatively charged particles compatible with cutaneous delivery; (ii) protect the encapsulated polyphenolic cargo within an amorphous lipid matrix; (iii) deliver antioxidant activity comparable to ascorbic acid while maintaining a non-cytotoxic and ecotoxicologically benign profile; and (iv) attenuate UVC- and H_2_O_2_-driven ROS accumulation in human melanocytes [9,10].

## 2. Results and Discussion

### 2.1. Phytochemical Fingerprint of the Jabuticaba Peel Extract

Targeted UPLC-MS/MS quantification (Tables S1 and S2) of 43 non-anthocyanin phenolic compounds and 6 anthocyanins revealed that the jabuticaba peel extract is characterized by a high density of benzoic acid derivatives, flavonols, and anthocyanins (Table 1 and Table 2, Tables S1 and S2). The hydroxybenzoic acid family is overwhelmingly represented by ellagic acid (~500 μg/g), accompanied by protocatechuic acid (~70 μg/g) and gallic acid (~20 μg/g). Flavonols are led by quercetin-3-rhamnoside and quercetin aglycone (~90 μg/g), and the quercetin-3-glucoside/galactoside fraction (~50 μg/g), with significant contributions from myricitrin (~30 μg/g), myricetin (~15 μg/g), and laricitrin (~10 μg/g). Among anthocyanins, cyanidin-3-glucoside dominates (~70 μg/g). Other relevant bioactive classes include phenylpropanoids, primarily p-coumaric acid (~35 μg/g).

Due to co-elution under the chromatographic conditions employed, quercetin-3-O-glucoside and quercetin-3-O-galactoside were quantified as a combined value (Q3G), as were syringetin-3-O-glucoside and syringetin-3-O-galactoside (S3G). Although the reference method resolves the quercetin pair separately [12], the isomers co-eluted in the present analysis and could not be integrated independently; for the syringetin pair, co-quantification is consistent with the reference method. This minor limitation does not affect the main conclusions, given that the phenolic profile is dominated by ellagic acid, quercetin-3-O-rhamnoside and quercetin.

Minor fractions of flavanones (naringenin), flavones (sinensetin), stilbenes (*trans* resveratrol and piceid), and dihydrochalcones (phloretin) (Table 1) were also identified, completing a fingerprint consistent with *Plinia cauliflora* and *Myrciaria jaboticaba* peels [4,13]. Although present in lower concentrations, these minor constituents may contribute to the overall antioxidant and biological activity of the extract, as synergistic interactions among structurally diverse phenolics have been reported for related extracts [14,15]. However, since no synergy assays were performed in the present study, the specific contribution of these minor compounds and their potential interactions with the major constituents remain to be experimentally confirmed.

This phenolic composition is consistent with a plausible multi-target antioxidant rationale, based on mechanisms previously reported for these compounds in isolated form. Ellagic acid has been shown to upregulate antioxidant defenses (CAT, SOD, GSH) and suppress UVB-induced MMP-1 expression and MMP-9 activity in human dermal fibroblasts [6]. Cyanidin-3-glucoside protects HaCaT keratinocytes against UVB-induced apoptosis by attenuating ROS, preserving mitochondrial membrane potential and suppressing caspase activation [6,7,16]. Quercetin and its glycosides, when delivered through NLC matrices, restore antioxidant enzyme activity and act as broadband photoprotective adjuvants. Caffeine has been described as an antioxidant whose activity depends on dose and delivery source, and has documented applications in skincare [5]. It should be noted that these mechanisms were characterized in previous studies using isolated compounds or different delivery systems and were not directly evaluated for NLC-JC in the present work; the phytochemical fingerprint reported here therefore provides a compositional basis for, rather than direct experimental evidence of, these mechanistic pathways.

**Table 2:** Concentrations and analytical identification data (UHPLC-MS/MS) of anthocyanins in the jabuticaba peel.

Compound	Concentration (µg g^−1^)	RT (Min)	ESI	Precursor (*m*/*z*)	Quantifier (*m*/*z*)	Qualifier (*m*/*z*)
Cyanidin-3-glucoside	72.987 ± 1.243	2.94	+	449	287	137
Pelargonidin-3-glucoside	0.365 ± 0.049	3.41	+	433	271	121
Delphinidin-3-glucoside	6.631 ± 0.335	2.59	+	465	303	229
Petunidin-3-glucoside	0.163 ± 0.077	3.17	+	479	317	302
Peonidin-3-glucoside	0.728 ± 0.048	3.51	+	463	301	286
Malvidin-3-glucoside	0.012 ± 0.005	3.62	+	493	331	315
Delphinidin-3-glucoside	6.631 ± 0.335	2.59	+	465	303	229

Anthocyanins were identified and quantified following Arapitsas et al. (2012) [17]. ESI: electrospray ionization mode; RT: retention time. Values of concentration in units of µg g^−1^ dried peel ± standard deviation over three replications in one sample (mean ± SD).

### 2.2. Physicochemical Design and Structural Characterization

The rational selection of the final NLC-JC platform is summarized in Figure 1. A four-arm pre-formulation screening was first conducted on empty nanostructured lipid carriers prepared with *Astrocaryum murumuru* butter, *Orbignya phalerata* oil and Kolliphor^®^ HS 15 at different lipid: surfactant ratios, applying a polydispersity index (PDI) threshold of <0.2 as the primary acceptance criterion for monodisperse colloidal systems (Figure 1A). Formulation F2 (2:1) was excluded due to severe polydispersity (PDI = 0.62, HD = 128.6 nm), while F1 (1:1), F3 (2:1) and F4 (2:1) satisfied the spec. Although F3 (PDI = 0.20, HD = 92.70 nm) formally qualified as a monodisperse system, its PDI value coincided precisely with the upper boundary of the pre-established acceptance threshold, leaving no safety margin against batch-to-batch variability or minor formulation perturbations. Such borderline behavior was considered a potential risk factor for colloidal robustness upon subsequent bioactive loading, and F3 was therefore not advanced to the second formulation tier, despite its nominal compliance with the monodispersity criterion. F1 produced the most monodisperse and smallest particles of the screening (PDI = 0.09; HD = 42.88 nm), positioning it in the optimal region of the PDI × HD design space.

To probe the robustness of the carrier under realistic loading conditions, F1 and F4—the two candidates with the most contrasting lipid: surfactant ratios—were advanced to a second formulation tier, in which the hydroethanolic jabuticaba peel extract, caffeine and a 10% NaDES-like microenvironment were co-incorporated (Figure 1B) [18,19,20]. F1 retained sub-spec monodispersity (PDI = 0.131) and a tight hydrodynamic diameter (HD = 56.08 nm), whereas F4 collapsed into a polydisperse coarse suspension (PDI = 0.536; HD = 157.8 nm). This marked increase in PDI for F4, from 0.18 at the initial empty-carrier screening to 0.536 after bioactive loading, indicates that F4’s failure occurred specifically upon incorporation of the extract, caffeine and NaDES-like phase, rather than reflecting a deficiency already present at the initial screening stage. Quantification of the relative deviation from the empty NLC baseline (Figure 1C) revealed a +198% PDI excursion for F4—well beyond the ≈100% threshold associated with colloidal destabilization—compared with only +46% for F1, evidencing a markedly higher loading tolerance of the 1:1 lipid: surfactant matrix. Taken together, these data demonstrate that F1 satisfies a Quality-by-Design space anchored on monodispersity, sub-100 nm particle size and tolerance to bioactive incorporation and was therefore selected as the final NLC-JC platform for all subsequent physicochemical, biological and ecotoxicological characterization.

Nanoparticle stability is critical for carrier performance, as storage temperature influences particle size, homogeneity, and dispersion, potentially causing aggregation or degradation [21]. NLC-JC stored at 0.5 °C and ~30 °C maintained stable hydrodynamic diameter (DH) values throughout the study (Figure 2A,B), indicating preserved physical stability. In contrast, samples stored at 37 °C showed a progressive increase in DH (Figure 2C), suggesting thermal instability [12,22]. PDI remained low at all temperatures, confirming homogeneous and reproducible suspensions. Zeta potential was stable and negative at lower temperatures, whereas at 37 °C it increased at day 15 and decreased at day 30, indicating temporary colloidal destabilization due to thermal effects or lipid matrix reorganization. Acidic pH was maintained under all conditions (Figure 2D), likely due to the NaDES-like component, which provides buffering capacity and chemical stability [19].

In this regard, it was determined that NLC-JC, as previously described by Mello et al. (2022), tends to remain more stable at lower storage temperatures due to reduced molecular mobility and lower lipid recrystallization within the lipid matrix under refrigeration [23]. In contrast, higher temperatures increase particle kinetic energy and lipid phase fluidity, favoring coalescence, aggregation, and hydrodynamic diameter changes, which compromise colloidal stability and system performance [24]. These findings reinforce that temperature is a critical factor for nanostructured system stability. Based on these results, storage of NLC-JC under refrigerated (0.5 °C) to controlled room-temperature (~30 °C) conditions, protected from prolonged exposure to temperatures at or above 37 °C—such as storage in vehicles, direct sunlight, or hot climates—is recommended to preserve colloidal integrity throughout the intended shelf life of the formulation.

### 2.3. Morphological Characterization and Ultrastructural Analysis of NLC-JC by Transmission Electron Microscopy

Transmission Electron Microscopy (TEM, Figure 3A,B) was performed to characterize the morphology of NLC-JC. Unlike DLS, in which the particles are in a colloidal suspension, in TEM, the sample undergoes processes that tend to destabilize or alter its morphology, such as contrast with osmium tetroxide (OsO_4_) [24]. The interaction of this contrast agent with the double bonds of the lipids present in the NLC can alter the surface charge of the nanoparticles, leading to aggregation, which causes the particles to appear larger in the images [24,25]. In addition to OsO_4_-induced aggregation, TEM samples were air-dried on Formvar-coated grids prior to imaging, a preparation step that removes the hydrated aqueous/surfactant corona measured by DLS and can cause partial collapse and flattening of the soft lipid nanoparticles on the grid surface, further increasing the apparent lateral diameter [24]. Since this hydrophilic corona is not electron-dense, TEM primarily resolves the electron-dense lipid core, so the combined effect of dehydration-induced spreading and OsO_4_-mediated aggregation, rather than a genuine increase in particle size, is considered the most likely explanation for the larger dimensions observed by TEM relative to DLS [25]. Considering this, the images revealed that the nanoparticles exhibit a predominantly spherical morphology, with average diameters of approximately 180 nm. The particles showed uniform dispersion, without signs of significant agglomeration, indicating good physical stability and compatibility between the lipids and the surfactant used. Additionally, a dense core structure was observed, suggesting efficient encapsulation [26]. The presented histogram (Figure 3C) indicates that the majority of particles observed in TEM have an average diameter of approximately 150–200 nm, corroborating the obtained images.

The structural organization of the lipid matrix was further investigated by X-ray diffraction (XRD) (Figure 3D). Both formulations displayed predominantly amorphous XRD profiles consisting of broad halos centered at approximately 20° and 40° 2θ. Quantitative deconvolution yielded crystallinity indices of 6.05% for Method A and 0.0% for Method B, confirming the essentially amorphous state of the lipid matrix. The two incorporation methods were not strictly equivalent: NLC-JC prepared by Method A retained a low-intensity residual reflection at approximately 12.2° 2θ, together with weaker reflections at approximately 20.4°, 21.7°, and 26.5° 2θ, whereas the Method B formulation displayed no sharp reflections, consistent with the most disordered matrix obtained when the extract is incorporated into the lipid phase (Table 3). The essentially amorphous character of both formulations is consistent with the formation of structural imperfections that increase the available space for bioactive encapsulation, and may improve loading capacity while facilitating controlled release and long-term stability of the phenolic compounds and caffeine within the carrier [20,27,28].

### 2.4. Cytotoxicity

For a potential cosmetic application, it is necessary that the carrier exhibits low toxicity at the concentrations of interest. Thus, the MTT assay evaluates cellular metabolic viability through the enzymatic reduction of the tetrazolium salt to formazan, which is insoluble and produced by mitochondrial dehydrogenases in viable cells; a decrease in absorbance indicates loss of viability [29]. As a result, it was found that the IC50 of NLC-JC was 2304 µg/mL (Figure 4), indicating that a high concentration of the technology is required to reduce cell viability by half. Practically, many studies in the biomedical and cosmetic fields adopt the 70% viability threshold (ISO 10993-5) to classify samples as non-cytotoxic; that is, reductions in viability of less than 30% do not constitute cytotoxicity [29,30,31,32].

Thus, the IC50 obtained for NLC demonstrates low toxicity and supports its cosmetic applicability, especially considering that the usual concentrations for topical use are substantially lower and the maintenance of ≥70% viability across the doses of interest meets the ISO 10993-5 criterion for non-toxicity. These results further underscore the safety of the system for proceeding with more specific functional tests and subsequent development stages. Since the MTT assay relies on mitochondrial dehydrogenase activity, and caffeine and polyphenols are known to directly modulate mitochondrial metabolism, a degree of metabolic interference with the MTT readout cannot be entirely ruled out, independent of any genuine effect on cell viability [31]. This possibility is nonetheless mitigated by the fact that the non-cytotoxic concentration range identified here coincides with the concentrations later shown to be biologically active in the antioxidant and regenerative assays, which are based on independent, non-MTT readouts, lending indirect support to the reliability of the estimated IC_50_. Even so, formal confirmation through an orthogonal, mitochondria-independent viability assay—such as LDH release or live/dead staining—would be a valuable step in future studies to definitively exclude such interference [31,32].

The negatively charged surface, the absence of cationic surfactants and the molecular dispersion of the polyphenolic cargo are likely contributors to this favorable safety profile, as cationic lipid systems have been associated with disproportionate membrane stress and oxidative damage in melanocytic models.

### 2.5. Ecotoxicological Evaluation and Developmental Impact of NLC-JC in the Zebrafish Embryo Model

With the increasing use of nanomaterials in consumer products, the growth of nanoecotoxicology has been driven, a field of toxicology focused on the potential toxicological effects that nanoparticles may induce in aquatic and terrestrial organisms when released into the environment [33,34,35]. Humans and animals can be exposed to nanoparticle-based products through contaminated water or air, or through the bioaccumulation and biomagnification of these nanomaterials [36,37,38,39]. Despite the development of “green” nanoparticles, data on toxicity in environmentally relevant species remain limited. Therefore, the Fish Embryo Toxicity (FET) test using zebrafish (*Danio rerio*) serves as a robust model for evaluating the safety profile of nanocarriers prior to large-scale application [40,41,42,43,44]. In this study, the activity of NLC-JC in the FET assay revealed that toxic effects are strictly concentration- and exposure time-dependent (Figure 5A).

The dose–response curve at 96 hpf showed a strong correlation between increasing concentration and embryo mortality (R^2^ = 0.98), allowing the calculation of an LC_50_ of 2013 µg/mL. Additionally, the LC_10_ was calculated at 776.6 µg/mL, representing the onset of significant lethality, while the LC_90_ was estimated at 5218 µg/mL, indicating the theoretical concentration required for near-total mortality (Figure 5B).

The obtained LC_10_ (776.6 µg/mL) aligns consistently with the experimental observations, where concentrations up to 843.75 µg/mL exhibited mortality rates comparable to the negative control. This validates the safety margin of the formulation at lower doses. Conversely, lethality escalated sharply above this threshold, with the three highest concentrations demonstrating a significant increase in mortality starting from 24 hpf (*p* < 0.001) (Figure 5C).

Despite the high mortality observed at the highest concentrations, a specific phenomenon was noted regarding the hatching rate between 24 and 48 hpf. A fraction of the embryos exposed to lethal doses managed to hatch, only to succumb immediately afterwards. The primary hypothesis for this behavior is the “desperate escape” response. Hatching in zebrafish is a finely regulated physiological process that depends on the integrity of a transient neuroendocrine circuit. This circuit, governed by hypothalamic Thyrotropin-Releasing Hormone (TRH), activates the hatching gland to secrete proteolytic enzymes (chorionases) that degrade the chorion. The completion of this process requires coordinated muscular activity of the embryo [45].

A plausible interpretation of this pattern is a ‘desperate escape’ response, in which severe toxic stress prematurely triggers hatching as an adaptive, rather than maladaptive, survival mechanism [46], consistent with reports that certain environmental stressors, in contrast to the hatching delay typically induced by many chemical toxicants [47,48], can instead accelerate hatching in zebrafish embryos. Under this hypothesis, individuals that hatched at 48 hpf may have already sustained damage sufficient to result in mortality shortly thereafter, which would be consistent with the high rate of post-hatching mortality observed in subsequent days. A complementary, and equally speculative, hypothesis involves transient antioxidant protection followed by systemic failure: the polyphenols and caffeine present in NLC-JC could plausibly offer temporary antioxidant protection during the high metabolic demand of hatching [49,50], given the hormetic, dose-dependent behavior reported for these compounds in the literature [51], before being overwhelmed at higher, more toxic concentrations. We emphasize that neither hypothesis was directly tested in the present study (e.g., via ROS quantification, chorionase activity, or neuroendocrine marker analysis), and we propose these as directions for future mechanistic investigation rather than as established explanations for the observed phenotype, particularly given the well-established utility of the zebrafish model for probing oxidative stress mechanisms in vivo [52].

Beyond the acute lethality endpoints, exposure to NLC-JC induced a spectrum of specific sublethal and teratogenic effects in surviving embryos, which were systematically recorded from 24 to 96 hpf. These developmental anomalies are visually documented in Figure 6A and quantified in Figure 6B–E. The photomacrographs reveal that structural malformations are strictly concentration-dependent. At the highest concentrations tested (1687.5 µg/mL and 3375 µg/mL), embryos exhibited severe deviations from normal development as early as 24 hpf. As highlighted by the red square in the 24 hpf panel of Figure 6A, treated embryos displayed a significant retardation characterized by the lack of tail detachment and undefined somite structures compared with the negative control, indicative of a disruption in the early segmentation phase.

As development progressed through 48, 72, and 96 hpf, specific malformations showed a statistically significant increase in incidence, particularly edema and metabolic disorders. Yolk Sac Edema (YSE, indicated by letter ‘a’) was the earliest and most frequent sign, followed by Pericardial Edema (PE, letter ‘d’) in later stages. As quantified in Figure 6B (YSE) and Figure 6D (PE), these conditions showed a highly significant increase (*p* < 0.0001) in prevalence at the two highest concentrations across all time points. These edemas are classic phenotypic signatures of osmoregulatory failure and potential cardiotoxicity, suggesting that high doses of NLC-JC may compromise epithelial barrier integrity or cardiac pumping efficiency [53]. Concurrently, distinct signs of metabolic impairment were statistically confirmed, specifically Yolk Sac Darkening (YSD, letter ‘b’) and Malabsorption Syndrome (MS, letter ‘c’). These effects, quantified in Figure 6C,E, point to an inability of the embryo to properly metabolize yolk reserves, leading to energy deficits that likely contribute to the observed growth retardation and eventual lethality [54].

In addition to these statistically significant parameters, other specific structural defects were identified through qualitative observation in Figure 6A, although they did not reach statistical significance in the overall population analysis. These included caudal malformations (blue arrow), evident at 48 and 96 hpf, suggesting disruptions in posterior axial elongation. Alterations in the notochord structure (green arrow) were also noted at 72 and 96 hpf; while not statistically different from the control in frequency, their presence at high doses represents potential damage to the primary signaling center for spinal development, as this structure serves as a key signaling center for the formation of the vertebral column and the central nervous system. Damage to this precursor structure can lead to severe secondary defects in axial development [55,56,57]. Furthermore, blood stasis (red arrow) was recorded at 72 hpf. Although this finding was qualitative and not statistically significant, the accumulation of blood cells due to insufficient circulation aligns with the cardiotoxic profile suggested by the pericardial edema, hinting at compromised hemodynamics in individual specimens exposed to extreme doses [52].

Despite the sublethal effects observed at the highest concentrations, a crucial finding was that at 96 hpf, organisms exposed to concentrations below 1687.5 µg/mL had restored developmental homeostasis, exhibiting phenotypes comparable to the negative control. Regarding mortality, the LC_50_ determined for NLC-JC was significantly higher than values previously reported for other nanomaterials in zebrafish studies, indicating that NLC-JC exhibits lower acute toxicity to the aquatic environment than compounds such as silver, zinc, other metals, and different lipid-based systems [19,23,57,58,59,60,61,62,63]. The assay, conducted according to OECD No. 236, confirmed that adverse effects were strictly dependent on concentration and exposure time [63]. The estimated LC_50_ at 96 h was approximately 2.01 g/L (2013 µg/mL). According to guidelines for acute hazard classification to the aquatic environment, substances with an LC_50_ greater than 0.1 g/L (100 mg/L) are not classified as acutely hazardous [64,65]. The obtained value for NLC-JC is on the order of 10^3^ mg/L, placing it well beyond the regulatory concern threshold and demonstrating a safety profile superior to many surfactants widely used in cosmetics. In practical terms, the concentrations intended for topical cosmetic applications utilize substantially lower fractions of the nanocarrier, and the inevitable dilution in aquatic ecosystems upon disposal makes environmental exposure to the toxic levels identified in this study highly unlikely [66,67].

It should be noted that the GHS acute hazard classification is based on lethality (LC_50_) alone and does not capture the sublethal effects (yolk sac/pericardial edema, malabsorption) observed at concentrations below this threshold (843.75–1687.5 µg/mL, Figure 6), which, as discussed above, remain well outside the range expected under realistic environmental exposure.

Mechanistically, the results suggest a typical hormetic response characteristic of the system’s constituents, such as caffeine and polyphenols: at low to medium levels, there is likely an attenuation of oxidative stress that supports physiological processes like hatching, whereas higher levels precipitate cumulative damage and lethality [67,68]. In the NLC-JC system, encapsulation and controlled release appear to mitigate exposure peaks, favoring beneficial biological activities while reducing the likelihood of reaching concentrations associated with adverse effects [69,70]. Thus, the finding of an LC_50_ of approximately 2.01 g/L, combined with the absence of increased mortality or malformations at ranges relevant for use, supports the ecotoxicological viability and the “safe-by-design” nature of the formulation under the intended application conditions.

### 2.6. Antioxidant Metabolism

#### 2.6.1. Antioxidant Capacity Determined by DPPH

Following the toxicity results, functional assays were conducted to evaluate the cosmetic potential of NLC-JC. Antioxidant activity is a key feature of nanostructured systems, as it reduces oxidative stress associated with UV radiation and pollution, both linked to skin aging [71]. This was assessed using the DPPH assay, which measures the ability of compounds to donate electrons or hydrogen atoms, reducing DPPH absorbance at 517 nm [72,73]. Ascorbic acid (AA) was used as the reference antioxidant [74]. NLC-JC showed ~50% antioxidant activity across all tested concentrations, with no significant differences among groups (Figure 7). Compared with AA, it exhibited similar activity at lower concentrations (13.18 and 26.36 µg/mL), indicating efficacy at low doses. While AA activity increased with concentration, NLC-JC reached a plateau, suggesting antioxidant saturation and possible controlled release of bioactives.

This behavior is characteristic of NLCs, which enable gradual and sustained release of bioactives, maintaining antioxidant activity over time and avoiding cytotoxic concentration spikes [75]. Rather than indicating saturation of antioxidant capacity, this plateau reflects the dynamic equilibrium between sustained polyphenolic delivery and radical neutralization at the colloidal interface, characteristic of NLC platforms and limiting cytotoxic concentration spikes while preserving long-acting redox modulation [76,77,78,79]. The synergistic combination of jabuticaba peel extract and caffeine enhances photoprotective and regenerative effects by neutralizing free radicals and stimulating endogenous antioxidant enzymes such as superoxide dismutase and catalase [80,81], supporting redox balance and protection against UV-induced damage and premature aging [82,83].

Importantly, the antioxidant-effective concentrations were non-toxic in the cell viability assay (Figure 4), supporting the safety of NLC-JC for cosmetic use. Overall, low toxicity combined with sustained antioxidant activity and regenerative potential highlights this system as a promising platform for skin repair formulations.

#### 2.6.2. Protective Effect of NLC-JC Against UVC-Induced Oxidative Stress

To explore additional applications of NLC-JC, ROS production was evaluated using a chemiluminescent probe. ROS are highly reactive oxygen-derived molecules, with the superoxide anion (O_2_^−^) as the main mediator of oxidative stress [83,84,85,86]. Under physiological conditions, superoxide dismutase (SOD) converts O_2_^−^ into O_2_ and H_2_O_2_, maintaining cellular homeostasis [7]. Reduced SOD activity due to aging, inflammation, mutations, or UV exposure leads to ROS accumulation, resulting in DNA damage, mitochondrial dysfunction, reduced ATP production, and cell death [7].

Ultraviolet (UV) radiation is a major inducer of oxidative stress and cellular damage [87]. The UV spectrum includes UVA (315–400 nm), UVB (280–315 nm), and UVC (100–280 nm), differing in energy and tissue penetration [88,89]. UVA (~90% of UV reaching Earth) penetrates the dermis and promotes ROS formation, DNA damage, inflammation, photoaging, and carcinogenesis [89,90,91,92], while UVB mainly affects the epidermis, causing direct DNA damage and increasing skin cancer risk [92,93,94]. UVC, although naturally filtered, is artificially generated and used as a model of extreme UV exposure, inducing severe damage such as DNA oxidation, pyrimidine dimers, and disruption of cell junctions [95,96,97].

UVC exposure rapidly induces loss of viability, apoptosis, DNA damage, and ROS accumulation, activating p53 responses and suppressing SOD activity, which amplifies oxidative stress [92,97,98,99]. In cell culture models lacking the stratum corneum, it causes strong dose-dependent cytotoxicity, making it useful for mutagenesis, DNA repair, and antimicrobial studies [100]. UVC also induces lesions similar to UVB, especially CPDs, triggering NER, p53 signaling, and cell cycle arrest. In fibroblasts, it enables controlled DNA damage studies, while in melanocytes, high doses can overcome melanin protection, supporting antioxidant and reparative approaches [101,102].

Using this model, regenerative mechanisms of NLC-JC were investigated. Average radiance over time (Figure 8A) showed low superoxide levels in untreated cells (Uns), while H_2_O_2_-treated cells (H_2_O_2_) exhibited increased O_2_^−^ production, validating the oxidative stress model. Under combined H_2_O_2_ and UVC exposure, NLC-JC-treated cells showed reduced absorbance compared with the H_2_O_2_ control, indicating decreased intracellular oxidative stress. This was confirmed by area under the curve analysis (Figure 8B), which demonstrated reduced total superoxide production. Mechanistically, this attenuation likely reflects the convergent contributions of ellagic acid (CAT/SOD/GSH upregulation; suppression of UVB-induced MMP-1 and MMP-9), cyanidin-3-glucoside (preservation of mitochondrial membrane potential and inhibition of caspase activation), protocatechuic acid (NF-κB suppression and MMP downregulation), quercetin glycosides (Nrf2-dependent endogenous antioxidant induction) and caffeine (hydroxyl radical scavenging, adenosine receptor cytoprotection and sunscreen adjuvant activity).

The literature supports these findings, showing that UVC-induced ROS generation, lipid peroxidation, mitochondrial damage, and apoptosis can be mitigated by phenolic antioxidants [101]. Similarly, antioxidant compounds have been shown to restore junctional proteins and reduce oxidative stress in UV-exposed keratinocytes [103,104]. Overall, NLC-JC demonstrates antioxidant and regenerative potential, supporting its use in skin repair and anti-aging formulations [102,103].

Confocal microscopy using a ROS-sensitive probe confirmed these effects. Untreated cells (Uns) showed preserved nuclei and cellular extensions with moderate fluorescence (Figure 8C). UVC exposure caused nuclear deformation and reduced extensions, indicating oxidative damage (Figure 8D). Combined UVC + H_2_O_2_ stress produced severe structural disruption and intense fluorescence, indicating high ROS levels (Figure 8E). In contrast, NLC-JC-treated cells showed reduced fluorescence and better-preserved morphology despite stress exposure (Figure 8F). These findings corroborate the antioxidant assays and align with reports on protective antioxidant nanosystems against UV-induced ROS generation [11,104].

## 3. Materials and Methods

### 3.1. Chemicals and Reagents

Ethanol, methanol, acetonitrile, chloroform, formic acid, betaine (≥98%), DL-malic acid (≥99%), 2,2-diphenyl-1-picrylhydrazyl (DPPH), bis-N-methylacridinium nitrate (lucigenin), 3-(4,5-dimethylthiazol-2-yl)-2,5-diphenyltetrazolium bromide (MTT), dimethyl sulfoxide (DMSO), osmium tetroxide, Formvar and 3,4-dichloroaniline were purchased from Sigma-Aldrich (St. Louis, MO, USA). Kolliphor^®^ HS 15 (macrogol 15 hydroxystearate) was obtained from BASF (Ludwigshafen, Germany). Anhydrous caffeine was obtained from Cromoline Química Fina (Diadema, São Paulo, Brazil). Dulbecco’s Modified Eagle Medium (DMEM), fetal bovine serum (FBS), penicillin-streptomycin, DAPI and CellROX™ Green were purchased from Thermo Fisher Scientific (Waltham, MA, USA). The SH-4 cell line was obtained from the Cell Bank of Rio de Janeiro (BCRJ, Rio de Janeiro, Brazil). The MTT reagent [3-(4,5-dimethylthiazol-2-yl)-2,5-diphenyl tetrazolium bromide] was obtained from Invitrogen (Thermo Fisher Scientific). Ultrapure water (Milli-Q, 18.2 MΩ cm; Merck Millipore, Burlington, MA, USA) was used throughout. Cupuaçu butter (*Theobroma grandiflorum*) and the oils of babassu (*Attalea speciosa*) and bacaba (*Oenocarpus bacaba*) were supplied by Amazon Oil (Ananindeua, Pará, Brazil) and used as received. The reference standards used for the identification and quantification of the phenolic compounds were of analytical or HPLC grade (purity ≥ 95%, as certified by the respective manufacturers). The supplier of each authentic standard is listed in the Supplementary Materials (Tables S1 and S2). Solvents used for the chromatographic analysis (methanol, acetonitrile and formic acid) were of LC-MS grade.

### 3.2. Plant Material, Botanical Identification and Regulatory Compliance

Jabuticaba fruits (*Plinia cauliflora* (DC.) Kausel) were collected in Sobradinho, Federal District, Brazil (15°37′42.373″ S, 47°49′55.408″ W), located in the Brazilian Savanna (Cerrado biome). Botanical identification was confirmed by the voucher specimen Reis, BC n.2, deposited at the Herbarium of the University of Brasília, where it remains as a reference for this study. All plant material was obtained in compliance with the applicable Brazilian regulations for access to genetic heritage, registered with the Sistema Nacional de Gestão do Patrimônio Genético e do Conhecimento Tradicional Associado (SisGen) under the codes A002D4B and A235193.

### 3.3. Preparation of the Jabuticaba Peel Extract

The fruits of *Plinia cauliflora* were collected in Sobradinho, Federal District (15°37′42.373″ S and 47°49′55.408″ W), located in the Brazilian Savanna. The peels were manually separated, lyophilized (L101 Liotop Lyophilizer, LABIOM São Carlos, Brazil), and stored at −40 °C. The extract used for NLC production was prepared from jabuticaba (*Plinia cauliflora*) peel according to Brito et al. (2022) [104]. The lyophilized peels were subjected to maceration with ethanol (96%-analytical grade) at a solid-to-solvent ratio of 1:5 (*w*/*v*), corresponding to 1 g of dry peels per 5 mL of solvent. The extraction was carried out in a single cycle under refrigeration (4 °C) for one week, protected from light. After this period, the extract was filtered under vacuum through qualitative filter paper (Whatman No. 1, 11 µm porosity) to remove solid residues. The resulting extract was stored at −20 °C until further use.

### 3.4. Phenolic Profiling by UPLC-MS/MS

The phenolic composition of the jabuticaba peel was determined by ultra-performance liquid chromatography coupled to tandem mass spectrometry (UPLC-MS/MS) using a Waters Xevo TQ (triple quadrupole) mass spectrometer. Non-anthocyanin phenolics were analyzed following the targeted multi-class workflow of Vrhovsek et al. (2012) [12], whereas anthocyanins were determined under the acidic conditions described by Arapitsas et al. (2012) [17].

Non-anthocyanin phenolics. Chromatography was performed on a Waters Acquity UPLC system (Milford, MA, USA) comprising a binary solvent manager, online vacuum degasser, autosampler and thermostatted column compartment. Analytes were resolved on a Waters Acquity HSS T3 column (1.8 µm, 100 mm × 2.1 mm) maintained at 40 °C. The mobile phase comprised water acidified with 0.1% formic acid (solvent A) and acetonitrile acidified with 0.1% formic acid (solvent B), delivered at 0.4 mL/min. Elution started at 5% B, ramped linearly to 20% B within 3 min, held isocratically at 20% B for 1.3 min, then rose linearly to 45% B by 9 min and to 100% B by 11 min; after a 2 min wash at 100% B, the system returned to the initial 5% B by 15 min. The injection volume was 2 µL, and the autosampler needle was flushed after each injection with 600 µL of weak wash (water/methanol, 90:10) and 200 µL of strong wash (methanol/water, 90:10). Samples were kept at 6 °C during the run.

Anthocyanins. Separation was carried out on a Waters Acquity UPLC BEH C18 column (1.7 µm, 2.1 × 150 mm) equipped with a BEH C18 precolumn (1.7 µm, 2.1 × 5 mm), maintained at 40 °C. The mobile phase consisted of water (solvent A) and methanol (solvent B), both acidified with 5% (*v*/*v*) formic acid, at a flow rate of 0.4 mL/min. The multistep linear gradient was: 95 to 60% A over the first 4 min, 60 to 45% A from 4 to 9 min, 45 to 5% A from 9 to 11 min, followed by a 3 min isocratic hold for column cleaning and 4 min of re-equilibration. The injection volume was 2 µL.

Mass spectrometry. Detection was performed on a Waters Xevo TQMS (Milford, MA, USA) equipped with an electrospray ionization (ESI) source. For non-anthocyanin phenolics, the capillary voltage was 3.5 kV (positive) and −2.5 kV (negative); source and desolvation temperatures were 150 °C and 500 °C, respectively, with cone gas and desolvation gas flows of 50 L/h and 800 L/h. Anthocyanins were analyzed in positive ionization mode with a capillary voltage of 0.5 kV, source and desolvation temperatures of 150 °C and 500 °C, and desolvation and cone gas flows of 1000 L/h and 20 L/h. Acquisition was performed in multiple reaction monitoring (MRM) mode with unit resolution, using the transitions optimized for each analyte (Table 1 and Table 2). A dwell time of at least 25 ms was applied to each MRM transition.

Data analysis. Data acquisition and processing were performed with MassLynx 4.1 and TargetLynx (Waters, Milford, MA, USA). Quantification relied on external calibration curves built with authentic standards, and peak assignment was confirmed by matching retention times and quantifier/qualifier ion ratios against the reference standards. The MRM transitions, retention times and method validation parameters (e.g., linear range, R^2^, LOQ) for the main compounds quantified in the extract are compiled in Table 1 and Table 2, Tables S1 and S2.

### 3.5. Preparation of the NaDES

The preparation of the Natural Deep Eutectic Solvent was carried out by combining betaine and malic acid (molar ratio of 1:1) with 49% water (*v*/*v*). The mixture was heated at 80 °C for 50–60 min until complete dissolution of the reagents.

### 3.6. Preparation of NLC-JC

NLC-JC was produced by the phase inversion temperature (PIT) method. The lipid phase, composed of 1.6% (*w*/*w*) solid lipid, 3.1% (*w*/*w*) liquid lipid and 4.7% (*w*/*w*) Kolliphor^®^ HS 15, was heated to 80 °C until complete melting. The aqueous phase, composed of 10% (*w*/*w*) NaDES, 5% (*w*/*w*) jabuticaba peel extract, 2% (*w*/*w*) anhydrous caffeine and ultrapure water to 100%, was heated separately to the same temperature.

The aqueous phase was added to the molten lipid phase under magnetic stirring at 300 rpm, and the coarse emulsion was subjected to high-shear homogenization (100 RPM for 3 min) across the phase inversion zone (70–75 °C). Nanoparticle formation occurred at approximately 75 °C with an emulsification time of 3–5 min, after which the system was rapidly cooled under running water to 25 °C. The batch size was 50 mL.

Two incorporation strategies for the jabuticaba peel extract were compared and are referred to throughout as Method A and Method B. In Method A, the extract was dissolved in the aqueous phase together with the NaDES and caffeine prior to emulsification, as described above. In Method B, the extract was dispersed directly into the molten lipid phase under stirring at 150 rpm for 3 min before addition of the aqueous phase. Both methods were characterized by XRD (Section 3.9).

### 3.7. Particle Size, Polydispersity, Zeta Potential and pH

Hydrodynamic diameter (HD, z-average), polydispersity index (PDI) and zeta (ζ) potential were determined by dynamic light scattering and electrophoretic light scattering on a ZetaSizer Nano ZS (Malvern Instruments, Malvern, UK), operating at 25 °C with a backscatter detection angle of 173°. Samples were diluted 1:10 (*v*/*v*). Each measurement comprised 12 runs, and results are reported as the mean ± SD of three measurements performed on each of three independently produced batches.

### 3.8. Colloidal Stability Study

Aliquots of NLC-JC were stored, protected from light, under three temperature conditions: 0.5 °C (refrigeration), 30 °C and 37 °C. On days 1, 7, 15 and 30, samples were equilibrated to 25 °C and analysed for HD, PDI, ζ-potential and pH as described in Section 3.8. Macroscopic appearance (phase separation, creaming, colour change) was recorded at each time point. Data are expressed as mean ± SD (*n* = 3 independent batches per condition).

### 3.9. X-Ray Diffraction

X-ray diffraction (XRD) patterns were recorded using a Rigaku MiniFlex 600 diffractometer (Rigaku Holdings Corporation, Tokyo, Japan) at the Green Nanotechnology Laboratory (NaVe, University of Brasília), with Cu Kα radiation (λ = 1.5406 Å), operated at 40 kV and 15 mA. Data were collected over a 2θ range of 5–80°, with a step size of 0.05° and a scan speed of 2° min^−1^. The NLC-JC formulations prepared by Methods A and B were analysed under identical conditions. In Method A, the extract was incorporated into the aqueous phase, whereas in Method B, it was added to the lipid phase.

The degree of crystallinity of each sample was quantified as the crystallinity index:

$$Cl\;=\;\frac{A_{cryst}}{A_{cryst}+A_{amorph}}\times 100$$

where *A_cryst_* is the integrated area of the crystalline reflections and *A_amorph_* is the integrated area of the amorphous scattering contribution. Each diffractogram was fitted with a combination of a polynomial baseline, broad Gaussian functions for the amorphous halos, and narrow Gaussian functions (FWHM ≤ 2°) for the crystalline reflections.

### 3.10. Transmission Electron Microscopy

Morphology and ultrastructural organization were assessed on a transmission electron microscope (JEOL JEM 1011, Tokyo, Japan) operated at an accelerating voltage of 80 kV. Samples were diluted 1:200 (*v*/*v*) in distilled water and deposited on copper grids coated with 0.4% (*w*/*v*) Formvar in chloroform. Grids were contrasted with osmium tetroxide vapour and air-dried before imaging at nominal magnifications above 10,000×. Particle diameters were measured on individual particles drawn from randomly selected fields across independent grids, using ImageJ version 1.54g (NIH, Bethesda, MD, USA).

### 3.11. Antioxidant Capacity—DPPH Radical Scavenging

The radical-scavenging capacity was determined according to Castangia et al. (2021) [9], with modifications. A 0.1 mM DPPH solution in ethanol was prepared fresh and protected from light. In a 96-well plate, 150 µL of sample (NLC-JC at 6.59, 13.18, 26.36, 52.73, 105.46, 210.93, 421.87 and 843.75 µg mL^−1^) was mixed with 150 µL of the DPPH solution. Plates were incubated in the dark at 25 °C for 30 min and absorbance was read at 517 nm on a Varioskan™ LUX multimode reader (Thermo Fisher Scientific, Waltham, MA, USA). Ascorbic acid (same concentration range) was used as the reference antioxidant. NLC-blank at matched total-solids concentrations, the free jabuticaba peel extract and free caffeine at the concentrations present in the corresponding NLC-JC dilutions were assayed in parallel. A sample blank (sample + ethanol, without DPPH) was prepared for every concentration to correct for the intrinsic absorbance and turbidity of the colloidal dispersion. Assays were performed in triplicate on three independent batches.

### 3.12. Cell Culture

SH-4 cells (human melanoma; ATCC CRL-7724) were cultured in DMEM supplemented with 10% (*v*/*v*) FBS and 1% (*v*/*v*) penicillin-streptomycin, and maintained at 37 °C in a humidified atmosphere containing 5% CO_2_.

### 3.13. Cytotoxicity—MTT Assay

Cytotoxicity was assessed in accordance with ISO 10993-5:2009 (Biological evaluation of medical devices—Tests for in vitro cytotoxicity), adopted here as the reference framework because no equivalent harmonized standard exists for topical cosmetic nanocarriers. SH-4 cells were seeded in 96-well plates at 5 × 10^3^ cells per well in 100 µL of complete medium and allowed to adhere for 24 h. Cells were then treated for 24 h with NLC-JC at 6.59, 13.18, 26.36, 52.73, 105.46, 210.93, 421.87, 843.75, 1687.5, 3375, 6750, 13,500 and 27,000 µg mL^−1^, with NLC-blank tested at the equivalent total-solids concentrations. Untreated cells served as the negative control. After treatment, the medium was replaced with 0.5 mg mL^−1^ MTT in DMEM and plates were incubated for 2 h at 37 °C. The formazan crystals were solubilized in 100 µL of DMSO and absorbance was read at 595 nm on a Varioskan LUX multimode reader (Thermo Fisher Scientific, Waltham, MA, USA). Viability was expressed as a percentage of the untreated control; samples reducing viability by less than 30% (i.e., viability ≥ 70%) were classified as non-cytotoxic, per ISO 10993-5.

### 3.14. UVC Irradiation and Induction of Oxidative Stress

Oxidative stress was induced by exposing the cells to ultraviolet C (UVC) radiation, followed by treatment with hydrogen peroxide (H_2_O_2_) as a positive oxidative stimulus. Cells were exposed to UVC radiation for 4 h and subsequently challenged with 26 mM H_2_O_2_ to enhance intracellular reactive oxygen species (ROS) generation. Untreated cells were used as the negative control, whereas UVC/H_2_O_2_-treated cells served as the positive oxidative stress control. To evaluate the antioxidant potential of the nanostructured lipid carrier, stressed cells were treated with 843.75 µg mL^−1^ NLC-JC for 24 h before subsequent analyses. NLC-JC was therefore applied strictly in a post-exposure (post-stress) treatment scheme, after UVC + H_2_O_2_-induced oxidative damage had already been established, rather than as a pre-treatment (prophylactic) or co-treatment (simultaneous) protocol. This design specifically models the regenerative/reparative cosmetic use case, evaluating the capacity of NLC-JC to counteract ROS accumulation and support cellular recovery after UV-induced oxidative insult, as distinct from a prophylactic photoprotection scenario. This experimental protocol was used for both the lucigenin-enhanced chemiluminescence assay and the CellROX™ Green confocal microscopy analysis.

### 3.15. Superoxide Anion Quantification by Lucigenin-Enhanced Chemiluminescence

SH-4 cells were seeded in 24-well plates at 1 × 10^5^ cells per well and subjected to the treatments described in Section 3.16. After treatment, 100 µL of lucigenin (250 µM in PBS) was added to each well and chemiluminescence was recorded on a Lumina XRMS Series III imaging system (Revvity Inc., Waltham, MA, USA). Results are expressed as average radiance (p s^−1^ cm^−2^ sr^−1^) as a function of time and as the corresponding area under the curve (AUC), computed by the trapezoidal rule. Assays were performed in technical replicates across three independent experiments.

### 3.16. Confocal Fluorescence Microscopy

SH-4 cells were seeded at 1 × 10^5^ cells per well on glass coverslips and subjected to the treatments described in Section 3.14. Cells were then incubated with 5 µM CellROX™ Green (Thermo Fisher Scientific) for 30 min at 37 °C, washed three times with PBS, and counterstained with 1 µg mL^−1^ DAPI. Confocal images were acquired on a Leica TCS SP5 confocal microscope (Leica Microsystems, Wetzlar, Germany) using a 63× oil-immersion objective lens, with excitation at 405 nm (DAPI) and 488 nm (CellROX Green).

### 3.17. Fish Embryo Acute Toxicity (FET) Test

The acute toxicity assay was performed in accordance with the Organization for Economic Co-operation and Development (OECD) Guideline No. 236 (2013) [65]. Zebrafish (*Danio rerio*) embryos were obtained from the Laboratory of Toxicological Genetics (G-Tox/UnB). The viable fertilized eggs were selected using a stereomicroscope (Stemi 2000 C; Carl Zeiss, Oberkochen, Germany), staged according to Kimmel et al. (1995) [105], and randomly distributed into 96-well microplates (one embryo per well), containing 200 µL of the test solutions. The assay was conducted in three independent replicates, totaling 60 embryos per experimental group.

The NLC-JC formulations were diluted in system water to obtain the following final concentrations: 52.87, 105.37, 211.12, 421.87, 843.75, 1687.5, and 3375 µg/mL. The negative control group consisted solely of system water (0 µg/mL). The plates were maintained in an incubator under controlled conditions, with a temperature of 26 ± 1 °C and a 12:12 h light/dark photoperiod.

Toxicity was monitored every 24 h up to 96 h post-fertilization (hpf). Lethality parameters evaluated included: egg coagulation, lack of somite formation, non-detachment of the tail, and absence of heartbeat. Additionally, sublethal and teratogenic parameters were recorded, specifically: yolk sac edema, pericardial edema, yolk sac darkening, and malabsorption syndrome, identified as deviations from the normal development described by Kimmel et al. (1995) [105].

### 3.18. Statistical Analysis

Mortality data were used to calculate the Median Lethal Concentration (LC_50_) using non-linear regression. Statistical analysis for sublethal parameters was performed using one-way analysis of variance (ANOVA) followed by Dunnett’s post-hoc test, considering values of *p* < 0.05 as statistically significant. Cell viability data were analyzed using nonlinear regression (dose–response inhibition) to determine the half-maximal inhibitory concentration (IC_50_) using GraphPad Prism 10.

## 4. Conclusions

The NLC-JC constitutes a stable and functional nanocarrier, developed from bioactives present in agro-industrial co-products incorporated into an Amazonian lipid matrix with a NaDES-like component, capable of preserving, targeting, and releasing phenolic compounds in a controlled manner. Physicochemical characterizations demonstrated homogeneous morphology with a hydrodynamic diameter of 56 nm by DLS and ~150–200 nm by TEM (the latter reflecting OsO_4_-induced surface rearrangement during sample preparation), narrow distribution, acidic pH compatible with anthocyanin stability, and a moderately negative surface charge, favoring interaction with the epidermal lipid barrier. Structural analysis by XRD indicated a predominantly amorphous profile, revealing a reduction in the crystallinity degree of the lipid matrix and suggesting greater encapsulation efficiency and higher molecular mobility of bioactives within the system.

In functional terms, NLC-JC exhibited antioxidant activity comparable to ascorbic acid at lower concentrations, and an effect in a cellular model exposed to UVC radiation, with reduced formation of reactive oxygen species. The safety profile confirmed the absence of cytotoxicity at relevant concentrations (IC_50_ 2304 µg/mL) in accordance with ISO 10993-5, and low acute toxicity in the FET assay with *Danio rerio*, reflected by an approximate LC_50_ of 2.01 g/L and by the absence of lethality at concentrations many times higher than those applicable to topical use. By integrating technological performance, safety, and sustainability, NLC-JC highlights the potential of green nanotechnology as a driver of innovation, while simultaneously promoting the reuse of agro-industrial residues (SDG 12), strengthening production chains based on biodiversity and the valorization of natural resources (SDG 15), stimulating clean and innovative industrial processes (SDG 9), and contributing to the development of safer products aligned with human well-being (SDG 3).

In summary, the results demonstrate that nanotechnology, circular bioeconomy, and biodiversity conservation can converge in a high-performance cosmetic platform with controlled risk and scalable potential, positioning NLC-JC as a promising candidate for future cosmetic and translational applications.

### Mechanistic Integration, Limitations and Outlook

Integrating the structural, cellular and ecotoxicological data, NLC-JC behaves as a multi-axis antioxidant nanocarrier whose performance is anchored in three complementary attributes. First, the amorphous, defect-rich lipid matrix derived from Amazonian butters and oils accommodates a chemically complex polyphenolic payload while limiting crystallization-driven expulsion typical of first-generation SLNs. Second, sub-200 nm dimensions, neutral-to-acidic surface pH and negative ζ-potential are physicochemical features associated with effective stratum corneum docking, intercellular lipid intercalation and transfollicular accumulation. Third, the bioactive payload itself constitutes a self-reinforcing antioxidant network in which ellagic acid, protocatechuic acid, cyanidin-3-glucoside, quercetin glycosides and caffeine cover complementary mechanistic axes. It should be noted that UPLC–MS/MS characterization of these phenolic markers was performed on the peel extract, prior to its exposure to the 80 °C thermal steps involved in NaDES preparation and lipid-phase emulsification; the major markers were not re-quantified in the final NLC-JC formulation. The antioxidant activity retained by the final formulation, comparable to ascorbic acid in the DPPH assay (Figure 7), provides indirect, functional evidence that antioxidant capacity is at least partially preserved after formulation, although it does not directly confirm the chemical integrity of each individual phenolic marker, nor exclude selective thermal degradation of more heat-labile compounds such as anthocyanins. Post-formulation UPLC–MS/MS quantification of the major phenolic markers in the final NLC-JC is identified as a direction for future work.

Some limitations should be acknowledged. The cellular assays were carried out in SH-4 human melanocytes, which capture the redox and pigment-cell axis but do not reproduce the full epidermal-dermal architecture; future studies in reconstructed human epidermis (RHE) and ex vivo skin will be required to evaluate stratum corneum penetration profiles, MMP-1/MMP-3 expression in primary fibroblasts and the kinetics of CPD repair. The UVC challenge, although valuable as a stringent mechanistic stressor, should be complemented by physiologically relevant UVA and UVB dosimetry. Ecotoxicological endpoints could be extended to chronic exposure and to a multi-species battery (algae, daphnids). Finally, dedicated encapsulation efficiency and in vitro release studies under accelerated photodegradation conditions will further consolidate the translation of NLC-JC into a regulatory dossier for dermocosmetic claims.

## Figures and Tables

**Figure 1:**
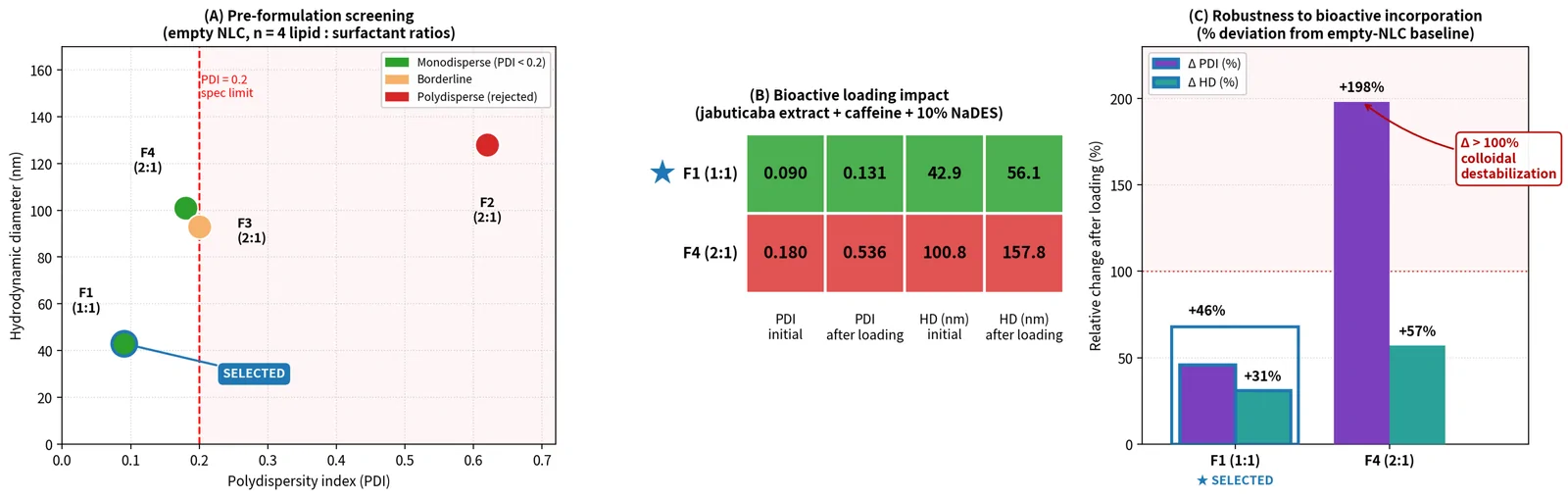
Pre-formulation roadmap leading to the rational selection of F1 as the final NLC-JC platform. (**A**) Pre-formulation screening of four lipid:surfactant ratios (F1, 1:1; F2–F4, 2:1) for empty nanostructured lipid carriers, plotted in the polydispersity index (PDI) × hydrodynamic diameter (HD) design space. The red-shaded region (PDI > 0.2) corresponds to polydisperse systems that fail the monodispersity acceptance criterion; circles are color-coded as monodisperse (green), borderline (amber) or rejected (red). F1 (blue-edged circle) was the only candidate combining sub-spec PDI (0.09) and sub-50 nm HD (42.88 nm); the leader line and “SELECTED” badge mark the formulation carried forward to panels (**B**,**C**). (**B**) Impact of bioactive loading (jabuticaba peel extract + caffeine + 10% NaDES) on F1 and F4, the two carriers carried forward from panel (**A**). Raw values of the four physicochemical descriptors (PDI initial, PDI after loading, HD initial, HD after loading) are annotated within the cells; cell shading distinguishes the selected formulation (green) from the non-selected one (red). The blue star (★) indicates the selected formulation. (**C**) Robustness analysis expressed as the relative percent deviation from the empty-NLC baseline after loading, for both PDI (purple bars) and HD (teal bars). The red-shaded region (Δ > 100%) marks the empirical threshold for colloidal destabilization. F1 (★ SELECTED, highlighted by the blue rectangular outline) showed only a +46% PDI and +31% HD deviation, while F4 exhibited a +198% PDI excursion—pinpointed by the red arrow—indicative of loading-induced colloidal collapse. Data are mean values (*n* = 3 independent batches per condition).

**Figure 2:**
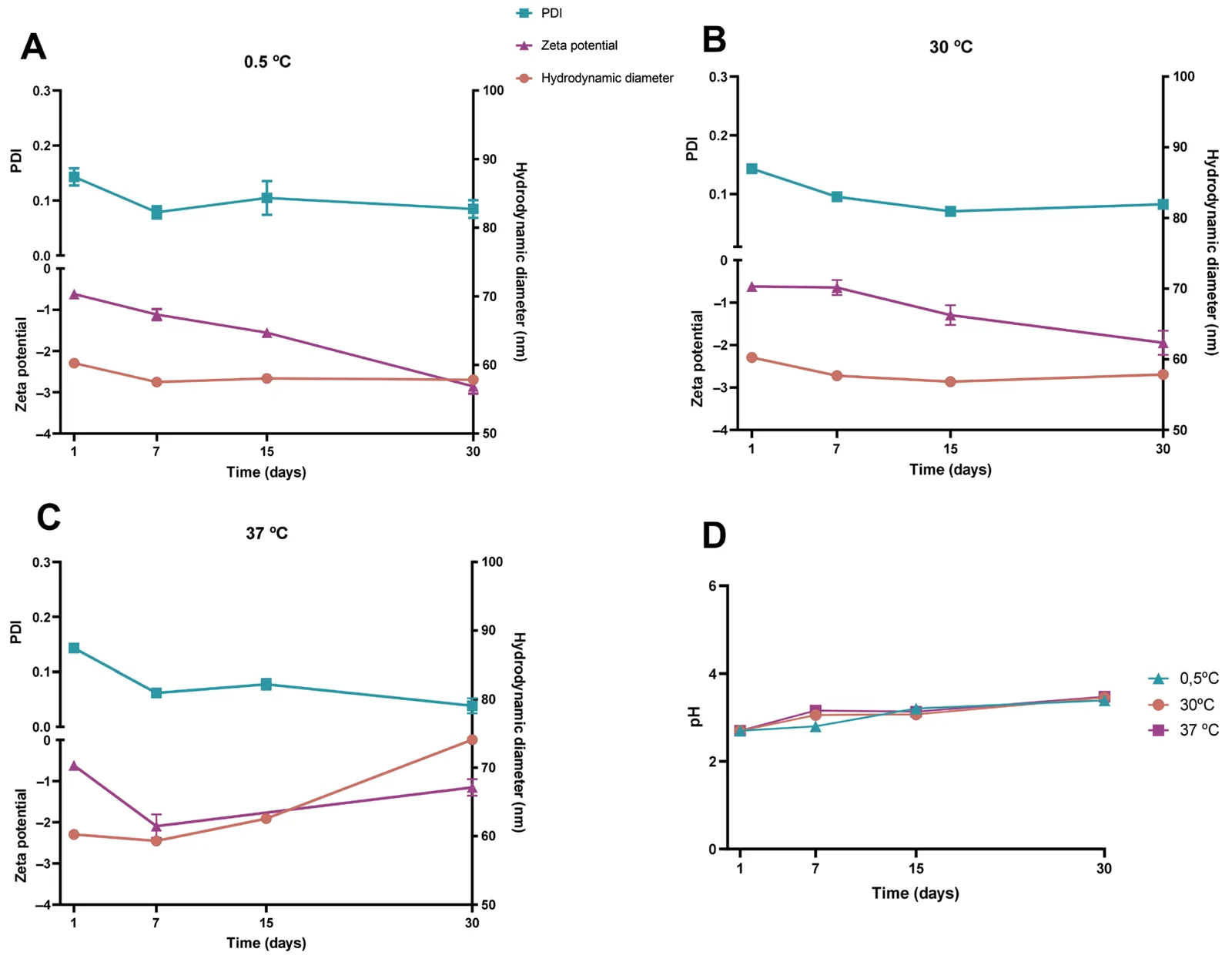
Time and temperature-dependent stability of NLC-JC. Hydrodynamic diameter (HD), polydispersity index (PDI) and ζ-potential measured by DLS at 25 °C on days 1, 7, 15 and 30 for samples stored at (**A**) 0.5 °C, (**B**) 30 °C and (**C**) 37 °C. (**D**) pH variation under the three storage conditions. Data are mean ± SD (*n* = 3).

**Figure 3:**
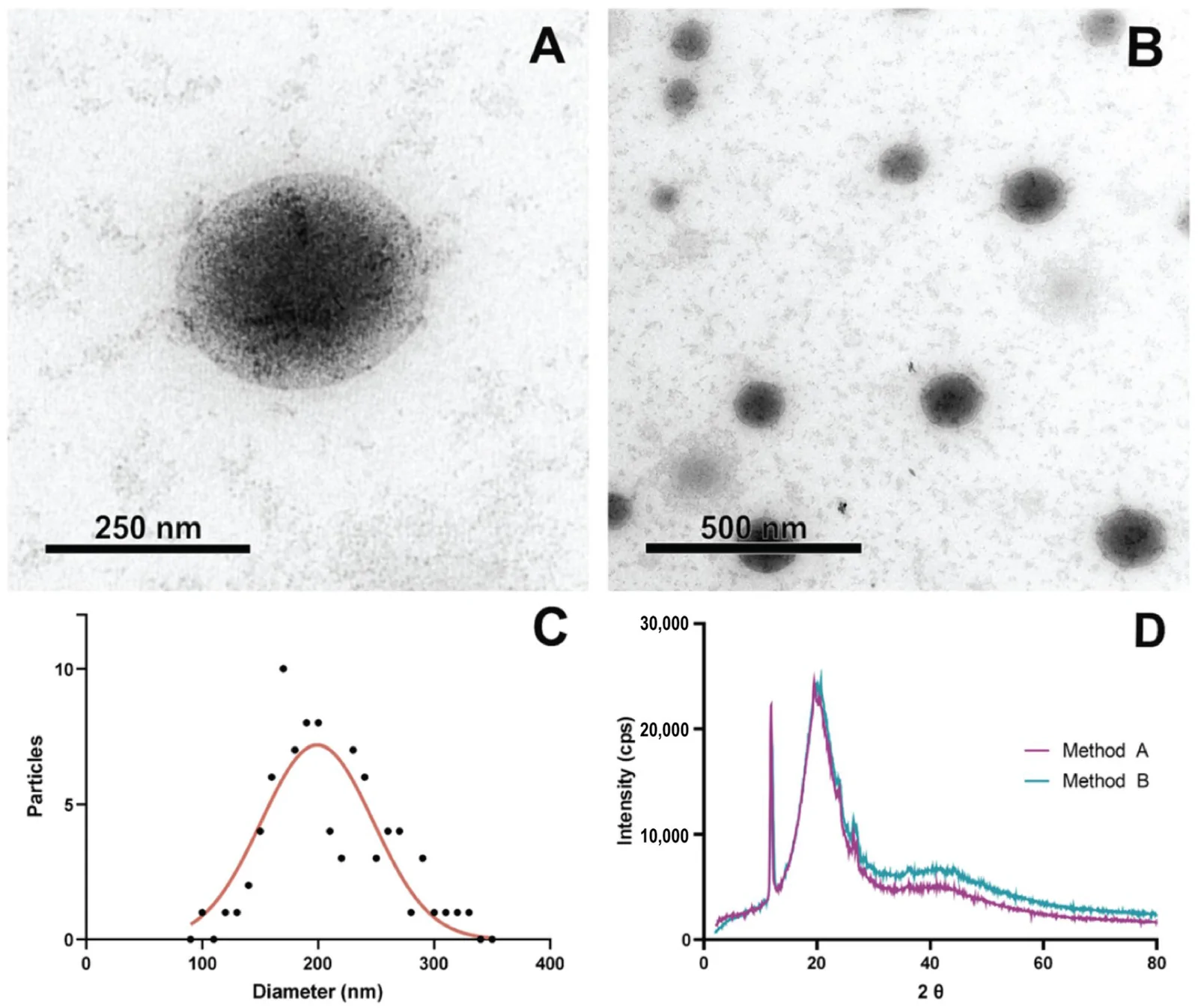
Structural and morphological characterization of NLC-JC. (**A**,**B**) Representative TEM micrographs at 250 nm and 500 nm scale bars showing spherical, electron-dense nanoparticles. (**C**) Particle-size distribution histogram derived from TEM analysis. (**D**) XRD patterns of NLC-JC prepared by Method A (extract in the aqueous phase) and Method B (extract in the lipid phase), showing predominantly amorphous profiles (broad halos centered at ~20° and ~40° 2θ) with low residual crystallinity. Method A retained a residual sharp reflection at approximately 12.2° 2θ (CI = 6.05%), whereas Method B displayed no sharp reflections (CI = 0.0%).

**Figure 4:**
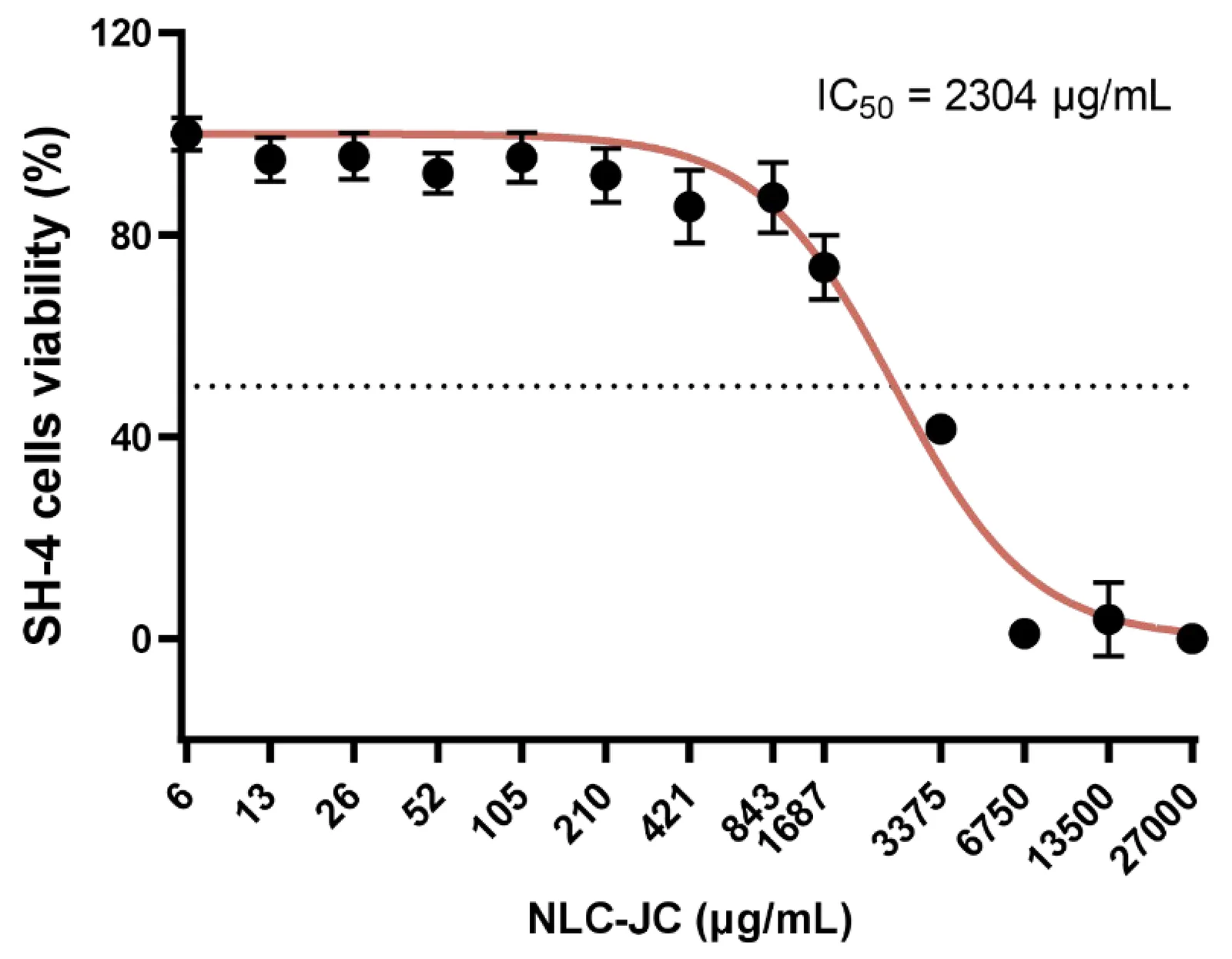
MTT-based cell viability of SH-4 melanocytes after 24 h exposure to NLC-JC. The estimated IC_50_ was 2304 μg mL^−1^. Data are mean ± SD (*n* = 3 independent experiments).

**Figure 5:**
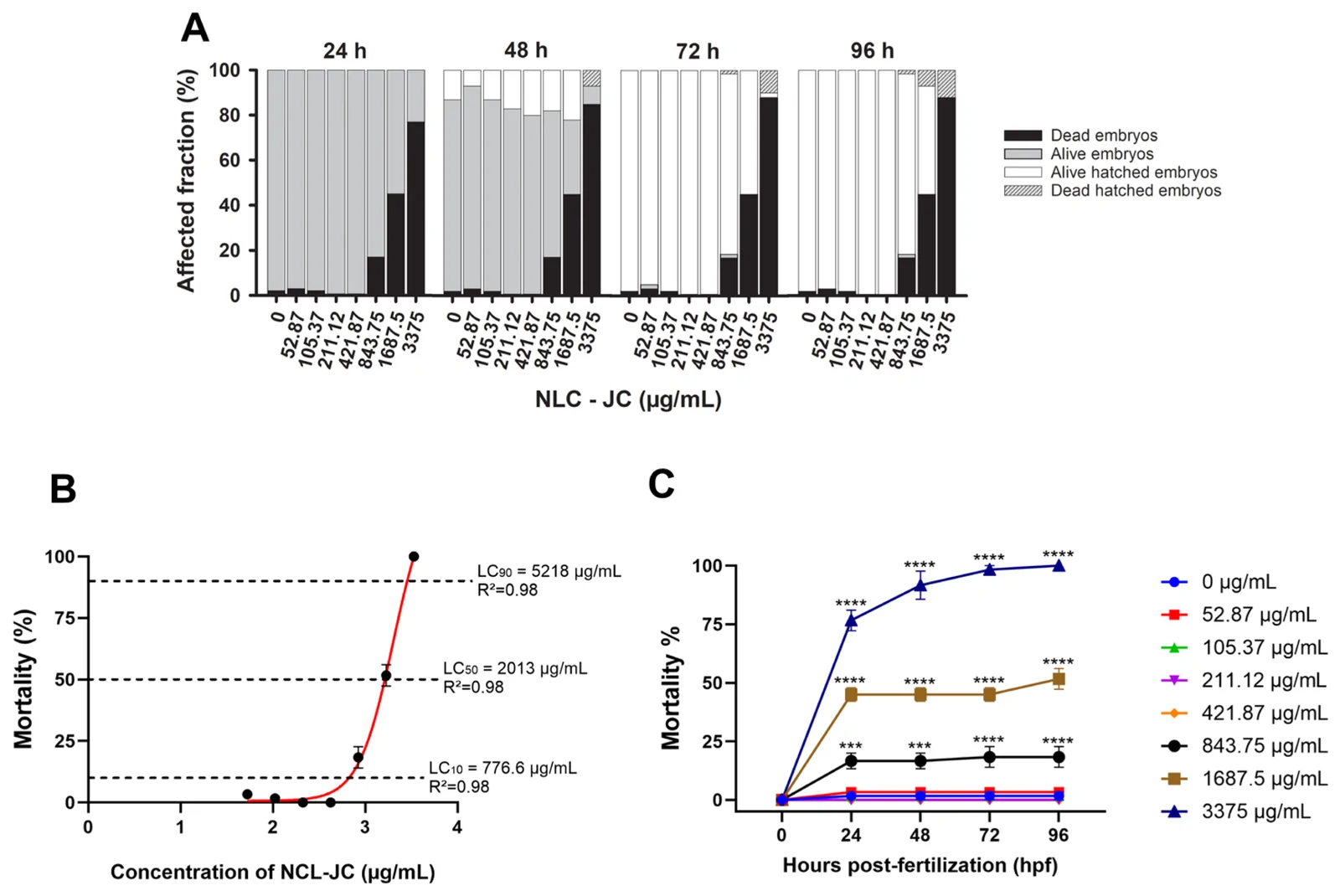
Acute lethality of NLC-JC in *Danio rerio* embryos (FET, OECD 236). (**A**) Cumulative mortality kinetics (%) assessed every 24 h up to 96 h post-fertilization (hpf) under different concentrations of NLC-JC. (**B**) Sigmoidal dose–response curve (non-linear regression) at 96 hpf, showing LC_10_ = 776.6 µg/mL, LC_50_ = 2013 µg/mL and LC_90_ = 5218 µg/mL (R^2^ = 0.98). (**C**) Cumulative mortality (%) over 24, 48, 72, and 96 hpf at different NLC-JC concentrations, expressed as mean ± standard error. Statistical significance relative to the negative control (0 µg/mL) was determined by two-way ANOVA followed by Dunnett’s post-hoc test: *** *p* < 0.001, **** *p* < 0.0001.

**Figure 6:**
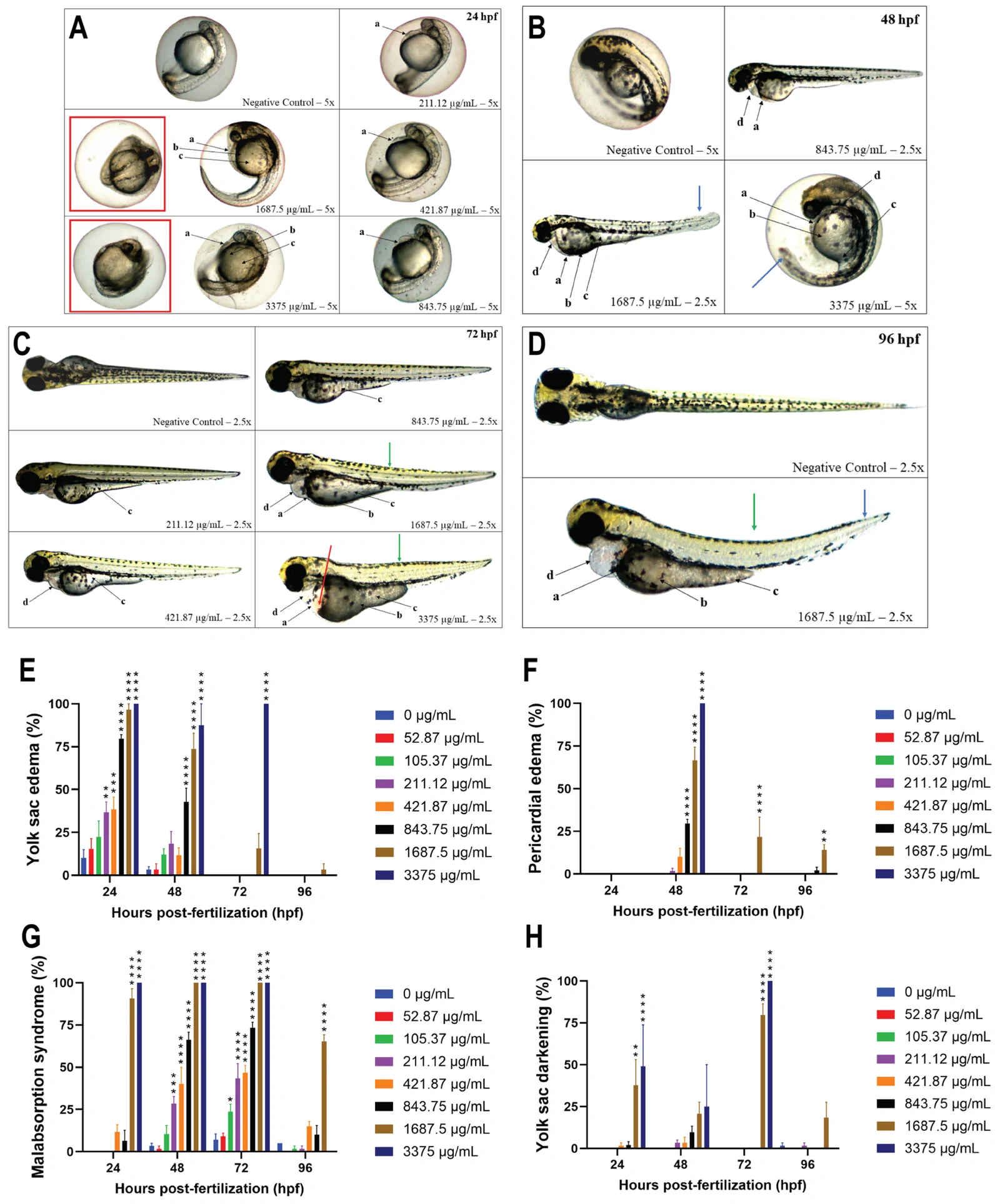
Sublethal effects observed over 96 h of exposure to NLC-JC in zebrafish (*Danio rerio*). (**A**–**D**) Photodocumentation of organisms showing developmental anomalies at different concentrations. The red square (24 hpf) highlights an embryo with developmental delay (altered tail separation). Black letters indicate the specific pathologies quantified in the graphs: (a) yolk sac edema, (b) yolk sac darkening, (c) malabsorption syndrome, and (d) pericardial edema. Colored arrows indicate qualitative systemic alterations: caudal malformation (blue arrow), notochord deformities (green arrow), and blood stasis (red arrow). (**E**) Yolk sac edema, (**F**) Pericardial edema, (**G**) Malabsorption syndrome, and (**H**) Yolk sac darkening. Quantitative analysis (%) of sublethal parameters expressed as mean ± standard error. * *p* < 0.05; ** *p* < 0.01; *** *p* < 0.001; **** *p* < 0.0001.

**Figure 7:**
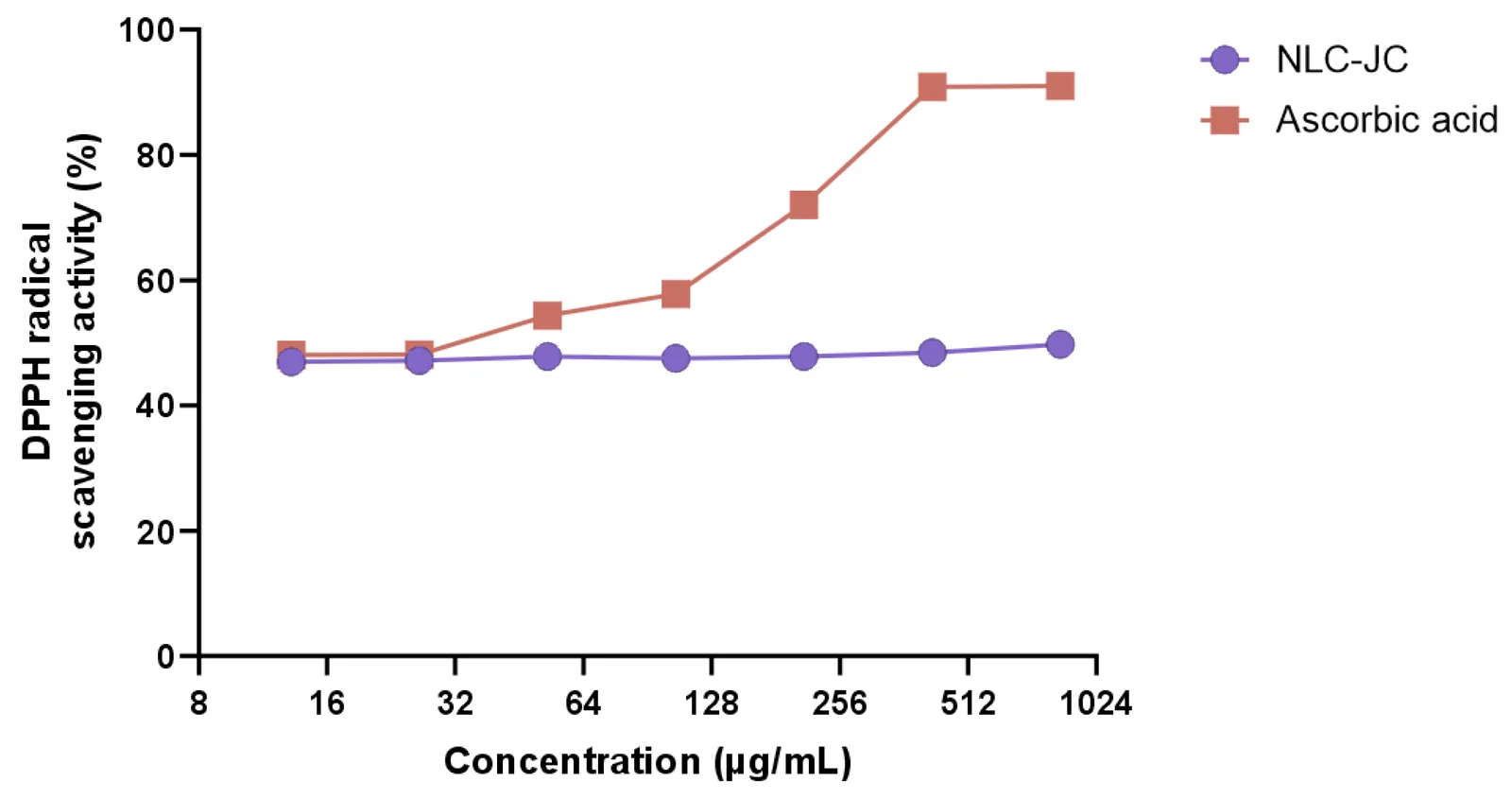
Antioxidant capacity determined via DPPH radical scavenging assay at different sample concentrations.

**Figure 8:**
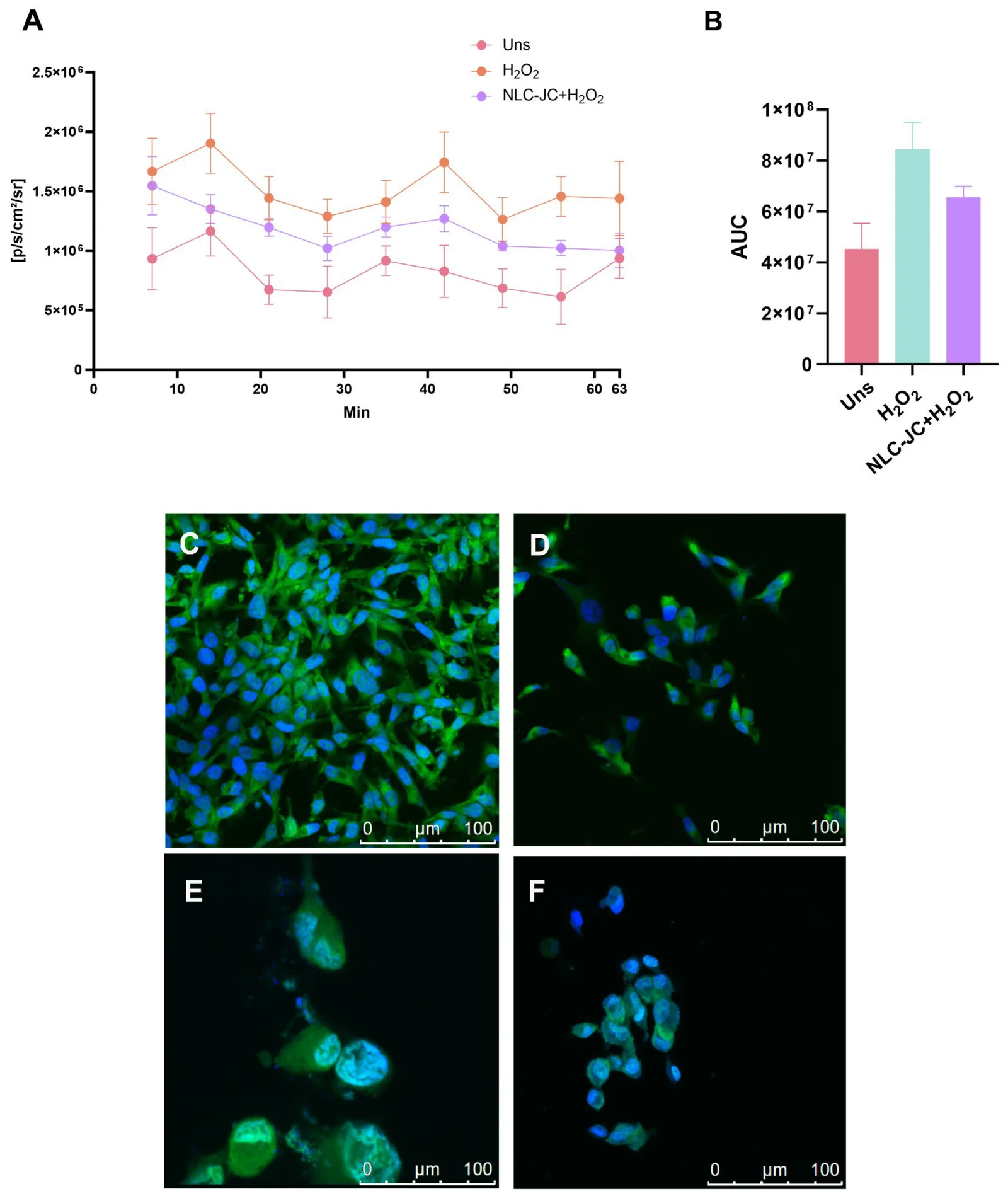
Antioxidant effect and cellular morphology analysis of NLC-JC under oxidative stress conditions. (**A**) Kinetic profile of superoxide anion production measured by lucigenin-enhanced chemiluminescence over 60 min in untreated cells (Uns), H_2_O_2_-induced cells (H_2_O_2_), and cells treated with NLC-JC (NLC-JC + H_2_O_2_). (**B**) Area under the curve (AUC) of chemiluminescence signals, indicating overall superoxide generation. (**C**–**F**). Representative fluorescence microscopy images of SH-4 cells showing cellular morphology and intracellular ROS-related fluorescence: (**C**) untreated cells (Uns), (**D**) UVC-induced cells, (**E**) UVC + H_2_O_2_-induced oxidative stress, (**F**) H_2_O_2_ + NLC-JC treatment. Scale bar = 100 µm.

**Table 1:** Concentrations and analytical identification data (UHPLC-MS/MS) of non-anthocyanin in the jabuticaba peel.

Compound	Concentration (µg g^−1^)	RT (Min)	ESI	Precursor (*m*/*z*)	Quantifier (*m*/*z*)	Qualifier (*m*/*z*)
***Benzoic acid derivatives***						
Vanillin	0.268 ± 0.025	4.07	+	153	93	125
Vanillic acid	2.343 ± 0.117	3.23	+	169	93	110
Gallic acid	20.083 ± 0.330	1.41	+	169	79	97
Protocatechuic acid	68.170 ± 2.238	2.12	+	153	81	91
Methyl gallate	6.883 ± 0.222	2.90	+	183	124	168
Syringaldehyde	0.117 ± 0.046	4.35	+	183	123	155
Ellagic acid	476.765 ± 19.727	4.38	−	301	145	185
***Coumarins***						
Daphnetin	0.007 ± 0.002	3.17	+	179	123	105
Scopoletin	0.016 ± 0.007	4.53	+	193	133	178
Esculin	0.006 ± 0.001	2.41	+	341	179	133
***Phenylpropanoids***						
p-Coumaric acid	36.303 ± 4.101	4.04	+	165	91	119
Cinnamic acid	10.050 ± 0.321	7.55	+	149	131	103
Caffeic acid	0.053 ± 0.010	3.19	+	181	145	117
Ferulic acid	0.678 ± 0.104	4.52	+	195	145	117
Chlorogenic acid	0.438 ± 0.009	2.76	−	353	191	—
Coniferyl aldehyde	0.055 ± 0.005	5.70	−	179	147	119
Coniferyl alcohol	0.026 ± 0.002	4.04	+	163	131	103
Sinapyl alcohol	2.837 ± 0.104	4.07	−	193	161	133
Trans-Coutaric acid	1.043 ± 0.065	2.85	−	295	163	—
***Stilbenes***						
Trans-Resveratrol	0.106 ± 0.004	6.56	−	227	185	143
Trans-Piceid	0.371 ± 0.019	4.27	−	389	227	185
***Dihydrochalcones***						
Phloretin	0.638 ± 0.082	8.23	−	273	167	123
Phlorizin	0.027 ± 0.005	6.22	−	435	273	167
*Flavones*						
Sinensetin	1.564 ± 0.055	9.84	+	373	343	312
Luteolin	0.403 ± 0.021	7.37	+	287	153	135
***Flavanones***						
Naringenin	2.299 ± 0.135	8.19	−	271	151	119
***Flavonols***						
Kaempferol	0.364 ± 0.059	8.45	+	287	153	165
Quercetin	83.678 ± 1.425	8.40	+	303	153	229
Taxifolin	1.708 ± 0.117	4.72	+	305	259	153
Isorhamnetin	2.835 ± 0.188	8.67	+	317	153	302
Myricetin	15.212 ± 0.264	6.07	+	319	153	245
Laricitrin	12.344 ± 0.254	7.46	+	333	318	219
Syringetin	3.802 ± 0.107	8.58	+	347	153	287
Quercetin-3-rhamnoside	90.922 ± 4.642	5.55	+	449	303	129
Myricitrin	30.922 ± 0.536	4.30	+	465	319	—
Quercetin-3-O-glucoside + quercetin-3-O-galactoside (as Q3G)	53.825 ± 1.739	4.50/4.37	+	465	303	229
Isorhamnetin-3-O-glucoside	3.098 ± 0.140	5.69	+	479	317	302
Syringetin-3-O-glucoside + syringetin-3-O-galactoside (as S3G)	1.003 ± 0.263	5.71	+	509	347	287
Rutin	0.024 ± 0.002	4.18	+	611	303	465
Dihydrokaempferol	0.685 ± 0.021	6.07	+	289	153	243
Quercetin-3-O-glucuronide	2.846 ± 0.133	4.45	+	479	303	229
Kaempferol-3-O-glucuronide	0.104 ± 0.007	5.45	+	463	287	165

Quercetin-3-O-glucoside and quercetin-3-O-galactoside were not baseline-resolved under the chromatographic conditions employed and were therefore quantified as a combined peak, expressed as quercetin-3-O-glucoside equivalents (Q3G). The same applies to syringetin-3-O-glucoside + syringetin-3-O-galactoside (S3G), consistent with the reference method [12]. Values in units of µg g^−1^ dried peel ± standard deviation over three replications in one sample (mean ± SD).

**Table 3:** Crystallinity index (CI) of the NLC-JC formulations.

Sample	CI (%)	Main Features (2θ)
NLC-JC (Method A)	6.05	Amorphous halos ~20°/~40° + sharp reflections ~12.2°, 20.4°, 21.7°, 26.5°
NLC-JC (Method B)	0.0	Amorphous halos ~20°/~40° only; no sharp reflections

## Data Availability

The original contributions presented in this study are included in the article/Supplementary Materials. Further inquiries can be directed to the corresponding author.

## Supplementary Materials

The following supporting information can be downloaded at https://www.mdpi.com/article/10.3390/ph19101517/s1: Table S1: MRM parameters, molecular formula, monoisotopic molecular weight and authentic standard of the phenolic compounds identified in the jabuticaba peel [12]; Table S2: Validation parameters (linear range, coefficient of determination r2, and limit of quantification LOQ) of key phenolic compounds quantified in the jabuticaba peel [17].
