# Supplementary material for: Nanocarrier System Based on Jabuticaba Peel Co-Products and Caffeine for Photoprotective and Regenerative Skin Applications
Source: Pharmaceuticals (Basel). 2026 Sep 25;19(10):1517. doi: 10.3390/ph19101517 (PMC13708402; doi:10.3390/ph19101517)
Supplement: Supplementary file 1 [file pharmaceuticals-19-01517-s001.zip › pharmaceuticals-4515772-supplementary.pdf]

SUPPLEMENTARY MATERIALS

Table S1. MRM parameters, molecular formula, monoisotopic molecular weight and authentic standard of the phenolic compounds identified in the jabuticaba peel.

Compound	Cone (V)	CE (V)	Molecular formula	MW (Da)	Authentic standard
Non-anthocyanin					
Vanillin	20	16	C8H8O3	152.14	Carlo Erba
Vanillic acid	18	14	C8H8O4	168.14	Carl Roth
Gallic acid	28	22	C7H6O5	170.12	Aldrich
Protocatechuic acid	24	20	C7H6O4	154.12	Carl Roth
Methyl gallate	32	22	C8H8O5	184.14	Fluka
Syringaldehyde	20	12	C9H10O4	182.17	Aldrich
Ellagic acid	52	34	C14H6O8	302.19	Fluka
Daphnetin	36	22	C9H6O4	178.14	Biomedicals
Scopoletin	34	20	C10H8O4	192.16	Fluka
Esculin	24	18	C15H16O9	340.29	Carl Roth
p-Coumaric acid	16	24	C9H8O3	164.16	Sigma
Cinnamic acid	16	12	C9H8O2	148.15	Aldrich
Caffeic acid	16	16	C9H8O4	180.16	Sigma
Ferulic acid	12	16	C10H10O4	194.18	Carl Roth
Chlorogenic acid	36	20	C16H18O9	354.31	TransMIT
Coniferyl aldehyde	22	14	C10H10O3	178.18	Aldrich
Coniferyl alcohol	12	10	C10H12O3	180.20	Aldrich
Sinapyl alcohol	14	10	C11H14O4	210.23	Aldrich
Trans-Coutaric acid	20	12	C13H12O8	296.23	Isolated
Trans-Resveratrol	38	18	C14H12O3	228.24	Isolated
Trans-Piceid	28	18	C20H22O8	390.38	Isolated
Phloretin	32	18	C15H14O5	274.26	TransMIT
Phlorizin	32	16	C21H24O10	436.41	TransMIT
Sinensetin	8	28	C20H20O7	372.36	Extrasynthese
Luteolin	52	32	C15H10O6	286.24	Sigma
Naringenin	32	18	C15H12O5	272.25	Carl Roth
Kaempferol	50	32	C15H10O6	286.23	Carl Roth
Quercetin	50	34	C15H10O7	302.24	Fluka
Taxifolin	20	14	C15H12O7	304.25	Sigma
Isorhamnetin	44	34	C16H12O7	316.26	Extrasynthese

Myricetin	44	32	$C_{15}H_{10}O_8$	318.23	Extrasynthese
Laricitrin	44	26	$C_{16}H_{12}O_8$	332.26	Extrasynthese
Syringetin	40	28	$C_{17}H_{14}O_8$	346.29	Extrasynthese
Quercetin-3-rhamnoside	16	10	$C_{21}H_{20}O_{11}$	448.38	Fluka
Myricitrin	14	10	$C_{21}H_{20}O_{12}$	464.37	Carl Roth
Quercetin-3-O-glucoside + galactoside (as Q3G)	18	12	$C_{21}H_{20}O_{12}$	464.38	Extrasynthese
Isorhamnetin-3-O-glucoside	16	14	$C_{22}H_{22}O_{12}$	478.41	Extrasynthese
Syringetin-3-O-glucoside + galactoside (as S3G)	16	14	$C_{23}H_{24}O_{13}$	508.43	Extrasynthese
Rutin	18	22	$C_{27}H_{30}O_{16}$	610.52	Extrasynthese
Dihydrokaempferol	18	18	$C_{15}H_{12}O_6$	288.25	TransMIT
Quercetin-3-O-glucuronide	20	18	$C_{21}H_{18}O_{13}$	478.37	Extrasynthese
Kaempferol-3-O-glucuronide	18	16	$C_{21}H_{18}O_{12}$	462.37	Extrasynthese
Anthocyanins					
Cyanidin 3-glucoside	28	30	$C_{21}H_{21}O_{11}$	449.39	Extrasynthese
Pelargonidin 3-glucoside	26	30	$C_{21}H_{21}O_{10}$	433.39	Extrasynthese
Delphinidin 3-glucoside	20	22	$C_{21}H_{21}O_{12}$	465.39	Extrasynthese
Petunidin 3-glucoside	28	30	$C_{22}H_{23}O_{12}$	479.42	Extrasynthese
Peonidin 3-glucoside	28	28	$C_{22}H_{23}O_{11}$	463.42	Extrasynthese
Malvidin 3-glucoside	28	30	$C_{23}H_{25}O_{12}$	493.44	Extrasynthese

Non-anthocyanin phenolics were analyzed following the targeted metabolomics method of Vrhovsek et al. (2012); anthocyanins following Arapitsas et al. (2012). Cone (V): cone voltage; CE (V): collision energy; MW: monoisotopic molecular weight. Quercetin-3-O-glucoside and quercetin-3-O-galactoside were quantified as a combined peak expressed as quercetin-3-O-glucoside equivalents (Q3G); syringetin-3-O-glucoside and syringetin-3-O-galactoside as syringetin-3-O-glucoside equivalents (S3G).

Table S2. Validation parameters (linear range, coefficient of determination r^2, and limit of quantification LOQ) of key phenolic compounds quantified in the jabuticaba peel.

**Compound**	**Linear range**	**R^2**	**LOQ**
Non-anthocyanin			
Gallic acid	80–60,000	0.998	160
Protocatechuic acid	60–20,000	0.996	120
Ellagic acid	400–20,000	0.994	800
p-Coumaric acid	6–10,000	0.990	12
Caffeic acid	6–10,000	0.994	12
Ferulic acid	1–8,000	0.995	2
Luteolin	100–8,000	0.990	200
Naringenin	2–2,000	0.991	4
Quercetin	4–10,000	0.995	8

Myricetin	8–20,000	0.997	16
Quercetin-3-O-rhamnoside	4–20,000	0.999	8
Rutin	8–20,000	0.998	16
Anthocyanin			
Cyanidin 3-glucoside	1065–266,250	0.993	355

Non-anthocyanins: Linear range: ng/mL; LOQ (pg): limit of quantification, defined as the concentration at which the quantifier transition presents a signal-to-noise ratio > 10 [12]. Anthocyanins: Linear range: µg/L; LOQ (µg/L): limit of quantification (signal-to-noise ratio = 10) [17]. R^2 from external calibration curves with authentic standards.
